# Nicotinic acetylcholine receptors and learning and memory deficits in Neuroinflammatory diseases

**DOI:** 10.3389/fnins.2023.1179611

**Published:** 2023-05-15

**Authors:** Valentina Echeverria, Cristhian Mendoza, Alex Iarkov

**Affiliations:** ^1^Facultad de Medicina y Ciencia, Universidad San Sebastián, Concepción, Chile; ^2^Research and Development Department, Bay Pines VAHCS, Bay Pines, FL, United States; ^3^Facultad de Odontologia y Ciencias de la Rehabilitacion, Universidad San Sebastián, Concepción, Chile

**Keywords:** nicotinic acetylcholine receptors, learning and memory, aging, cotinine, neurogenesis, posttraumatic stress disorder, traumatic brain injury

## Abstract

Animal survival depends on cognitive abilities such as learning and memory to adapt to environmental changes. Memory functions require an enhanced activity and connectivity of a particular arrangement of engram neurons, supported by the concerted action of neurons, glia, and vascular cells. The deterioration of the cholinergic system is a common occurrence in neurological conditions exacerbated by aging such as traumatic brain injury (TBI), posttraumatic stress disorder (PTSD), Alzheimer’s disease (AD), and Parkinson’s disease (PD). Cotinine is a cholinergic modulator with neuroprotective, antidepressant, anti-inflammatory, antioxidant, and memory-enhancing effects. Current evidence suggests Cotinine’s beneficial effects on cognition results from the positive modulation of the α7-nicotinic acetylcholine receptors (nAChRs) and the inhibition of the toll-like receptors (TLRs). The α7nAChR affects brain functions by modulating the function of neurons, glia, endothelial, immune, and dendritic cells and regulates inhibitory and excitatory neurotransmission throughout the GABA interneurons. In addition, Cotinine acting on the α7 nAChRs and TLR reduces neuroinflammation by inhibiting the release of pro-inflammatory cytokines by the immune cells. Also, α7nAChRs stimulate signaling pathways supporting structural, biochemical, electrochemical, and cellular changes in the Central nervous system during the cognitive processes, including Neurogenesis. Here, the mechanisms of memory formation as well as potential mechanisms of action of Cotinine on memory preservation in aging and neurological diseases are discussed.

## 1. Introduction

Learning and memory are not only essential tools for adaptation and survival but give us identity and the opportunity to build our individual and collective advancement based on previous experiences. During a learning experience, we gather sensorimotor, spatial, and emotional information and combine them with similar memories stored in the past.

Learning and memory comprise acquisition, memory consolidation, recall, and reconsolidation or extinction. During memory consolidation, temporary memory transforms into long-term memory (LTM), the mechanism by which acquired memories gain stability or become stronger over time and resistant to interference (Brashers-Krug et al., 1996). At the beginning of the last century, Richard Semon introduced two main theories of memory consolidation, the engram and synaptic plasticity theories. The concept of engrams describes the neural bases for memory storage and hypothesizes that an experience activates a specific group of hippocampal cells that undergo stable biochemical and structural changes to become engrams or memory traces (Semon and Simon, 1921; Thompson, 1976; Thompson, 2005; Thompson, 2013).

Donald Hebb stated that transforming a temporary memory into LTM required a concurrent activation and strengthening of pre-and postsynaptic neurons, a concept broadly supported by the scientific community (Kandel and Schwartz, 1982; Kandel, 2001; Day and Sweatt, 2011; Mayford et al., 2012; Kandel et al., 2014; Day et al., 2015; Asok et al., 2019).

Although still in its initial steps, a deeper understanding of brain functions opens the gates to an unknown and uncertain world where artificial intelligence arrives as an ethical and technological challenge rapidly impacting our society in its social and philosophical foundations. On the other side, however, the same advances promise to improve the quality of life of a crescent population of elderly people worldwide.

In the last decades, when life expectancy grew steadily, cognitive dysfunction during aging has devastated individuals and their significant ones.

The cognitive decline during aging is characterized by hippocampal dysfunction induced by neuronal loss and synaptic loss. These losses seem to be caused by the activation of immune cells, provoking neuroinflammation, mitochondria abnormalities, oxidative stress, decreased autophagy, and diminished Neurogenesis (Echeverria, 2016; Echeverria et al., 2016a; Yanai et al., 2022; Bathini et al., 2023; Chen et al., 2023; Choi and Tanzi, 2023; Dranovsky et al., 2023; Wang et al., 2023; Zhou et al., 2023). Thus, every year, the battle to preserve or augment cognitive abilities in the elderly becomes more critical for humanity. This review will discuss the potential mechanism of the alkaloid Cotinine, a positive cholinergic modulator, in preventing memory loss during aging and disease.

## 2. Memory mechanisms and engram cells

Episodic memory tells us what happened, when, and where it occurred (Reis et al., 2016). Unfortunately, this type of memory is the first to disappear during aging or dementia (Nyberg et al., 1996a). Dr. Brenda Milner and her team discovered that the hippocampus is the central brain region required to form episodic LTMs (Nyberg et al., 1996b). Almost 30 years later, Bliss and Lomo described long-term potentiation (LTP), a cellular mechanism of LTM (Bliss and Collingridge, 1993). In agreement with Hebbian’s theory, LTP entails increased presynaptic transmission, a concomitant rise of postsynaptic excitability, and the depolarization of the N-methyl-D-aspartic acid receptors (NMDARs). Simultaneous activation of these inotropic receptors at the presynaptic and postsynaptic terminals enhances the influx of calcium ions into neurons, triggering calcium waves and stimulating the expression of genes involved in memory acquisition and consolidation (Nicoll and Malenka, 1999).

The introduction of LTP as a molecular mechanism of memory was followed by the description of a second mechanism named long-term depression (LTD), both considered fundamental mechanisms of memory (Nicoll and Malenka, 1999; Castellano et al., 2001; Frey, 2001; Nakazawa et al., 2004). Finally, the spike-timing-dependent plasticity mechanism shaped the view of other cellular processes underlying both the acquisition and consolidation of LTM (Bi and Poo, 1998; Feldman, 2012).

The role of LTP and LTD *in vivo* in learning and memory is an unfinished discussion; however, the scientific community agrees that the formation and consolidation of episodic memory involve new mRNA transcription and protein synthesis, two processes also required in the late phases of LTP (Feldman, 2009; Alberini and Kandel, 2014; Fioriti et al., 2015). In addition, LTM consolidation demands metabolic, electric, and structural changes that reinforce particular synaptic connections and consolidate memories by establishing distinct neuronal networks.

As stated above, at the beginning of the last century, Dr. Semon introduced the term engram to describe the neural bases for memory storage (Thompson, 1976, 2013; Tonegawa et al., 2015, 2018; Josselyn and Tonegawa, 2020). According to Semon, an experience activates a specific group of cells that undergo stable biochemical and structural changes to become engrams or memory traces (Semon and Simon, 1921; Thompson, 1976, 2005, 2013; Josselyn and Tonegawa, 2020). Semon’s ideas were ignored during his lifetime; however, in the past decade, the access to transgenic animal models and techniques such as optogenetics, electrophysiology, and *in vivo* brain imaging, permitted to identify of the engram neurons activated during learning and forming part of the neural circuits involved in LTM consolidation and retrieval. Previous studies have shown that consolidation of LTM requires synaptic plasticity changes, which demand cellular changes in the metabolism, neurotransmission, and cell structure to reinforce synaptic connections establishing distinct neuronal networks (Izquierdo and Medina, 1997; Kandel, 1997; Kandel, 2001; Bailey et al., 2004). New technologies including gene editing (Nihongaki et al., 2015), single-cell RNA sequencing (Landry et al., 2013), *in vivo* transcriptional imaging (Das et al., 2018), and the optical control of epigenetic marks, have permitted to investigate posttranscriptional mechanisms controlling LTM storage and retrieval during aging and brain disease (Rajasethupathy et al., 2012; Landry et al., 2013).

In agreement with Semon’s theory, the engram neurons seem to acquire a stable biochemical and cellular state after activation, which is susceptible to being reactivated to retrieve, inhibit, or modify a particular memory content. For example, after the acquisition of a conditioned contextual memory, in the absence of a conditioned stimulus, it is possible to induce a false fear memory and conditioned fear reaction by pairing the activation of engram cells with exposure to a new context (Tonegawa et al., 2015, 2018; Josselyn and Tonegawa, 2020).

In the fifties, more than 73 years ago, a visionary scientist, Karl Spencer Lashley, defined the main concepts of memory storage as engrams throughout the cortex forming a sophisticated network connecting specialized brain cells preserving the representation of memories in the brain (Bruce, 2001). Additional technical advances such as the dual-eGRASP (green fluorescent protein reconstitution across synaptic partners; Choi et al., 2018) permitted the visualization of synaptic changes between engram cells during memory formation. These studies showed potential regional neuronal sites for memory processing and storage, including the hippocampus, ventromedial prefrontal cortex, anterior temporal lobe, amygdala (Choi et al., 2021), and posterior cortical areas with engrams in the dentate gyrus participating in spatial navigation memory. More importantly, they reported that partially nonoverlapping engram ensembles in the dentate gyrus encoded different paths during spatial navigation in rodents (Kim et al., 2018; Choi et al., 2021; Choi and Kaang, 2022; Roy et al., 2022).

### 2.1. The receptor mosaic hypothesis for memory trace formation

Other hypotheses were later developed, further enriching our understanding of memory mechanisms, including the receptor mosaic hypothesis for memory trace formation. This hypothesis postulated that receptors form complex aggregates in the plasma membrane. These aggregates would develop as the result of specific interactions between the receptors promoted by the input of neurotransmitters a neuron receives because of the cognitive process. These receptors form a distinctive mosaic of receptors stabilized by adaptor proteins. The expression and transport of the adaptors would be regulated by the neurochemical codes produced by the summation of neuronal inputs codifying a particular memory (Agnati et al., 1982, 2003, 2004; Zoli et al., 1996; Fuxe et al., 2007).

Also, the order and timing of spikes between pre-and postsynaptic neurons called spike-timing-dependent plasticity (STDP), defines the sign and magnitude of the synaptic plasticity event (Bell et al., 1997; Markram et al., 1997; Bi, 2002; Kepecs et al., 2002; Dan and Poo, 2004; Tazerart et al., 2020). For example, in pyramidal neurons, when a presynaptic spike precedes the postsynaptic one within a limited time, LTP is triggered; if the inverse occurs, the result will be LTD (Bi and Poo, 1998).

Recently, Dr.Tazerart used a spine STDP protocol with two-photon glutamate uncaging (pre) paired with postsynaptic spikes in the cortical layer V of pyramidal neurons to investigate how timing influences LTP in mice (Tazerart et al., 2020). They found that pre-post pairings triggered timing-dependent LTP (t-LTP), neck contraction in the activated spine, and increased synapse strength. On the contrary, post-pre pairings elicited timing-dependent LTD (t-LTD) and decreased synaptic strength without inducing a detectable change in spine structure (Tazerart et al., 2020). Also, they found that triggering t-LTP in clustered spines (<5 μm apart) enhanced LTP by an NMDAR-dependent increase of calcium inside the spine. The NMDAR-dependent t-LTD was interrupted due to the activation of clustered spines but normalized when the distance between spines was at a distance >40 μm. The authors concluded that synaptic cooperativity prevented t-LTD by extending the period permitted for STDP and LTP induction, thus increasing the chance of synaptic plasticity changes underlying LTM to occur (Tazerart et al., 2020). Later, an impairment of STDP was detected in the hippocampus of an APP/PS1 mouse model of AD, overexpressing the Amyloid beta peptide (Aβ) (Garad et al., 2021).

Initially, memory storage was understood as a linear and unidirectional process, transmitting memories from the hippocampus to cortical regions that, in turn, send them for storage in other cortical areas. Recently, Gilboa and Moscovitch (2021), argued that memory might have interconnected representations (abstract semantic, sensory, episodic, and emotional) (Gilboa and Moscovitch, 2021; Wing et al., 2021).

Memories are susceptible to modification after retrieval and reconsolidation, a characteristic exploited to extinguish fear memory using exposure therapy (Nijdam and Vermetten, 2018; Fazel et al., 2020; Gandelman et al., 2022; Wilker et al., 2023); However, fear memory usually reinstates, after exposure to events stimulating the unextinguished components of the memory even long time after extinction (Schiller et al., 2008; Lin et al., 2011; Ishii et al., 2012; Yang et al., 2012).

A better understanding of the detailed effects of fear and different therapies on engram cell’s connectivity and signaling factors (Han et al., 2022; Lee et al., 2023; Wilker et al., 2023) may guide the finding of effective and safe treatments for conditions associated with fear memory, such as PTSD and phobias.

In addition, we may consider non-synaptic transmission, glial cells such as the astrocytes, the extracellular space organization, and volume transmission (Vargova and Sykova, 2014). Similarly, other actors such as non-synaptic receptors (Vizi et al., 2010) metal ions (Chang, 2017), lipids (Wan et al., 2020), nucleic acid (Shao et al., 2020; Jana et al., 2023), and hormones (Hsu et al., 2021; Ghosh-Swaby et al., 2022; Harrington et al., 2022; Krinke et al., 2022; Su et al., 2022; Szczurowska et al., 2022; Wu et al., 2022; Zhang et al., 2022; Zhu D. et al., 2022; Kling et al., 2023; Lacasse et al., 2023) that simultaneously influence the brain response to external stimuli as well as internal abstract conceptualizations, to generate new or modified memories or ideas.

## 3. Role of the nAChRs in learning and memory

The cholinergic system is critical in keeping high-order cognitive processes in the brain by modulating inflammation, neurotransmitter release, and the expression of many essential genes participating in learning and memory. The cholinergic system involves the muscarinic AChRs (mAChRs) and nicotinic AChRs (nAChRs) in neuronal and non-neuronal cells (Echeverria et al., 2016b; Maurer and Williams, 2017; Mendoza et al., 2018a; Fuenzalida et al., 2020; Galvin et al., 2020; Yousuf et al., 2023). During the formation of new memories, ACh, an endogenous ligand of the AChRs, stimulates inhibitory and excitatory synapses in the hippocampus, adding flexibility to the brain networks during complex behaviors (Haam and Yakel, 2017). In addition, ACh modulates STDP by mechanisms influenced by the firing rate of neurons (Sjostrom et al., 2001) and synapse location and distance to the excitatory inputs (Kampa et al., 2006; Letzkus et al., 2006; Kampa et al., 2007; Letzkus et al., 2007; Froemke et al., 2010).

The hippocampus and the medial prefrontal cortex are brain regions, vastly populated with α7nAChRs, playing a fundamental role in acquiring, storing, and recalling working and episodic memory. In addition, one of its ligands, acetylcholine (ACh), influences STDP and adds flexibility to the brain networks during complex behaviors. The pentameric α7nAChRs are ligand-gated ion channels expressed pre- and postsynaptically in neurons (Cordero-Erausquin et al., 2000). The activation of presynaptic nAChRs promotes neurotransmitter release (Girod et al., 2000; Schilstrom et al., 2000), while activation of postsynaptic nAChRs controls cell signaling cascades supporting learning and memory, such as the PKA/CREB pathway (Kenney and Gould, 2008). In addition, the activation of the nAChRs induces LTP (Matsuyama et al., 2000; Matsuyama and Matsumoto, 2003).

The nAChRs are expressed in the dopaminergic, GABAergic, glutamatergic, norepinephrinergic, and serotonergic neurons (Albuquerque et al., 2009; Hernandez-Lopez et al., 2013; Wallace and Bertrand, 2013). When located on the inhibitory GABAergic, and excitatory glutamatergic neurons, the nAChRs regulate the circuits supporting episodic memory and the associated emotional content (Karadsheh et al., 2004). The nAChRs induce calcium to flow into the cell, triggering signaling cascades promoting synaptic plasticity (McKay et al., 2000; Karadsheh et al., 2004). New evidence suggests that the nAChRs play a specific role in reconsolidating long-term object memories (Wideman et al., 2022).

The nAChRs in neuronal, immune, and glial cells mediate the anti-inflammatory effect. Also, the nAChRs stimulate the expression of genes codifying for transcription factors involved in brain plasticity, such as the brain-derived neurotrophic factor (BDNF) and the cAMP response element-binding protein (CREB), and protein kinases such as the protein kinase A (PKA), the extracellular-signal-regulated kinases (ERKs), the protein kinases C (PKC), the phosphoinositide 3-kinases (PI3K), the protein kinase B (Akt), and the Calcium/calmodulin-dependent protein kinase II (CaMKII). These factors promote the expression of synaptic proteins, including Synapsin I/II, postsynaptic density protein-95 (PSD95), and Synaptophysin (Garad et al., 2021).

### 3.1. The nAChRs, engrams, and fear memory

Studies of the engrams in the hippocampus found that contextual fear memory formation involved enhanced connectivity between CA3 -and CA1 engram cells, which showed an increased number and size of synaptic spines. Furthermore, they concluded that higher synaptic connectivity between engram cells from adjacent brain regions of the hippocampus is part of the contextual fear memory formation mechanism.

It is known that the genetic background and environment influence a subject’s high-order cognitive abilities and behavior.

Further studies, using genetically modified mice not expressing the α7, β3, or β4 nAChRs subunits, showed that these mice have normal contextual and cued fear conditioning. This evidence suggests that these receptors are not required for fear memory acquisition and storage in mice (Davis and Gould, 2007). Recently, Lotfipour and colleagues (2013) showed that α2 nAChR KO mice showed regular contextual fear conditioning and no effect of nicotine on contextual or trace fear conditioning. However, sex-related differences in their role in fear conditioning were later discovered (Mohammadi-Farani et al., 2022). For example, Semenova et al. reported that male β4 KO mice presented deficits in cued fear conditioning while females did not (Semenova et al., 2012).

Our results working with the modulator of the nAChRs, Cotinine, acting as a positive allosteric modulator of the α7nAChRs enhanced contextual fear memory extinction without altering the acquisition and short-term retention of fear memory in a nAChR dependent-manner (Lotfipour et al., 2013). Based on th current evidence, we have postulated that cotinine-induced enhancement of extinction is mediated by increased neuronal plasticity and neurogenesis in rodents (Echeverria et al., 2017; Sadigh-Eteghad et al., 2020; Bastianini et al., 2021; Raghu et al., 2021). Few years ago, Sadigh-Eteghad et al. (2020) found that chronic cotinine ameliorated learning and memory in aged animals in the MWM and novel object recognition (NOR) tasks (Sadigh-Eteghad et al., 2020), and also increased the expression of (a) antioxidant factors such as the superoxide dismutase (SOD), (b) synaptic proteins (PSD95, GAP-43, and synaptophysin), (c) neurotrophic factors brain-derived neurotrophic factor (BDNF), and neural growth factor (NGF), while decreasing cytokines (TNFα and IL1β) in the hippocampus of aged mice (Sadigh-Eteghad et al., 2020). The α7nAChRs antagonist MLA blocked cotinine effects, in agreement with our results on mice models of PTSD (Echeverria et al., 2016a; Mendoza et al., 2018a; Oliveros-Matus et al., 2020).

A study of the effect of nicotine on contextual fear conditioning in β2 knockout mice did not show enhancement or impairment of fear conditioning compared to wild-type mice (Davis and Gould, 2007). Another study found slight contextual fear-conditioning deficits in the β2 knockout mice (Wehner et al., 2004; Wehner and Radcliffe, 2004). Interestingly, aging seems to change the role of β2 subunits in fear conditioning, as young β2 KO mice showed normal contextual and cued fear conditioning, in contrast to aged β2 KO mice that were impaired in both forms of fear learning (Caldarone et al., 2000). In this respect, β2 nAChR subunits may compensate for the decreased NMDA receptors observed in the aged mice, a function absent in the young β2 KO mice. This study also showed that while α2, α7, and β2 KO mice showed regular trace fear conditioning, β2 KO mice did not show the enhancing effect of nicotine over trace conditioning (Davis and Gould, 2007; Lotfipour et al., 2013).

On the other hand, new studies have shown that transcriptional regulation is also essential in modulating experience-directed behavior. In addition, epigenetic mechanisms can regulate nAChRs expression during memory acquisition through gene methylation and acetylation of chromatin. Changes in nAChRs expression should affect behavior and susceptibility to stress (Morrison et al., 2022).

## 4. Changes in cholinergic function in neurodegenerative conditions

The cholinergic neurotransmission occurs throughout the entire brain, controlling critical physiological processes such as attention, learning and memory, stress response, wakefulness, and sensory information acquisition (Nees, 2015). For many years, cholinergic dysfunction has been recognized as the leading cause of synaptic deficits leading to cognitive impairment in AD. Several studies using imaging and immunohistochemical approaches have shown degeneration of the cholinergic neurons in the basal forebrain (Jurgensen and Ferreira, 2010; Foucault-Fruchard and Antier, 2017) and a progressive loss of cholinergic receptors in the synaptic sites a primary cause of cognitive impairment during aging and neurodegenerative conditions (Bubser et al., 2012; Yakel, 2013; Okada et al., 2022; Yousuf et al., 2023).

Based on the accumulated evidence, it is reasonable to propose that due to the powerful anti-inflammatory effects of the α7nAChR, its reduced expression in AD and other neurological conditions is a crucial factor inducing synaptic deficits and neuroinflammation (Egea et al., 2015; Heneka et al., 2015; Lykhmus et al., 2015).

The abnormalities initially express as mild cognitive impairment (MCI) that may or not advance to dementia. The leading cause of dementia, Alzheimer’s disease (AD), is characterized by the progressive degeneration of cholinergic neurons in the basal forebrain and loss of nAChRs (Tiwari et al., 2019; Lacalle-Aurioles and Iturria-Medina, 2023; Tryon et al., 2023). In animal AD models, memory loss is characterized by neuronal mitochondrial dysfunction and deficits in the axonal transport of proteins and organelles. These defects are mainly attributed to the aggregation of the microtubule protein Tau and amyloid-beta peptides (Aβ). The intracellular accumulation of oligomeric forms of Aβ, is followed by the intracellular accumulation of aggregated forms of hyperphosphorylated Tau (Echeverria et al., 2002, 2004, 2007; Echeverria and Cuello, 2002). These abnormalities trigger the release of cytokines, such as the Transforming Neurotrophic Factor α (TNFα), neuroinflammation, oxidative stress, and mitochondrial dysfunction. This results in energy deficits and neuron cell death in brain regions involved in learning and memory (Bathini et al., 2023; Teoh et al., 2023; Wang et al., 2023). Activation of the α7nAChRs inhibits cytokine release by microglia, macrophages, astrocytes, and T cells in the CNS (McKay et al., 2007; Hernandez and Dineley, 2012; Sadigh-Eteghad et al., 2016; Morioka et al., 2018). Inhibition of cytokines release prevents neuroinflammation and neuronal damage induced by toxic protein aggregates in AD. Counteracting neuroinflammation and oxidative stress reduces Aβ peptides aggregation, abnormal tau phosphorylation, and conversion into intracellular neurofibrillary tangles (Echeverria et al., 2016a). Furthermore, re-establishing brain homeostasis reduces the metabolic deficit, oxidative stress, and mitochondria dysfunction (Echeverria et al., 2016b). Similar positive effects are expected by enhancing nAChRs activity to reduce neuroinflammation and neurodegeneration in other neurological conditions presenting a decrease in nAChRs, such as PD (Majdi et al., 2018; Iarkov et al., 2020).

## 5. Cognitive impairment during traumatic brain injury

TBI is commonly associated with the deterioration of executive functions, spatiotemporal memory, and recognition memory in the absence of dementia due to stress response and vascular pathology (Brett et al., 2021; Lippa et al., 2021; Steward et al., 2022; Williams et al., 2022).

Also, loss of cognitive abilities in TBI patients results from using drugs to diminish pain or anxiety, such as using anesthetics during and opioids after surgery, respectively (Stratmann et al., 2010; Lin et al., 2012; Devick et al., 2020). In addition, with few exceptions such as sertraline, most antidepressants, anxiolytic, and antipsychotics drugs radiation or chemotherapy, or new treatments such as ketamine investigated for treating PTSD, drug-resistant depression, diminishes cognitive abilities (Yasar et al., 2008; Mitchell and Turton, 2011; White et al., 2013; Hohman et al., 2015). Triggering events associated with a vulnerable genetic background work together to produce neuroinflammation and progressive deterioration of cognitive functions by reducing synaptic plasticity and the connectivity between brain regions supporting healthy cognitive functions (Rowan et al., 2007).

A recent study shows that single whole-body exposure to radiation decreased Neurogenesis, synaptic potentiation, and learning abilities in adult mice (Miry et al., 2021). Moreover, 2 months post-exposure, mice still showed plasticity deficits compared to sham-irradiated and age-matched mice. Unexpectedly, 6-, 12-, and 20 months after radiation, mice showed enhanced synaptic potentiation, spatial learning, and adult neurogenesis (Miry et al., 2021). Furthermore, synaptic plasticity and spatial learning remained enhanced 20 months post-exposure, indicating a life-long influence on plasticity and cognition from a single exposure to radiation in young adulthood. The authors speculated that galactic radiation exposure might alter brain health and cognitive function during or after a long trip in space (Miry et al., 2021). To our best knowledge, the mechanisms underlying the long-term effects of radiation on cognitive abilities and their impact on older individuals are not entirely understood.

## 6. Memory loss during aging

The preservation of learning and memory capabilities is one of the most challenging and needed healthcare goals to achieve, considering the growth of the aged population (Dinius et al., 2023). Various clinical trials have found numerous changes in the brain that may explain the negative impact of unhealthy aging on cognitive abilities. Cognitive impairment during aging is associated with hormonal deficits (Amore, 2005; Henderson, 2011; Winczyk et al., 2022), inflammation (Engler-Chiurazzi et al., 2023; Teoh et al., 2023; Zhou et al., 2023), epigenetic modifications affecting gene expression and adult neurogenesis (Zocher and Toda, 2023), and changes in the cholinergic, glutamatergic, dopaminergic, and histaminergic neurotransmission (Gasiorowska et al., 2021). These changes reduce synaptic and brain plasticity and memory loss (Shen and Barnes, 1996; Echeverria et al., 2017; Kumar et al., 2019b; Gasiorowska et al., 2021; Sanchez-Sarasua et al., 2022).

Incorporating brain imaging, optogenetic, and electric brain recording techniques in aging studies has allowed us to understand better the compensatory mechanisms preserving brain functionality and evaluate different therapeutic strategies. Magnetic resonance imaging (MRI) studies have shown that structural changes, such as the decline in the volume of the prefrontal areas and hippocampus during aging, can predict a person’s cognitive performance (Raz et al., 1997; Morrison and Baxter, 2012). New approaches tested to maintain or improve cognitive abilities during aging include hormonal supplementation (Foster et al., 2003), magnetic brain stimulation, oxygen therapy (Yang C. et al., 2023), cognitive stimulation using computer-assisted programs (Curlik 2nd and Shors, 2013; Clemenson and Stark, 2015; Nebot et al., 2022), guided exercise, music and dance therapy (Thumuluri et al., 2021; Aguinaga et al., 2022; Ambegaonkar et al., 2022; Kennedy et al., 2022; Li et al., 2022; Rodziewicz-Flis et al., 2022; Vega-Avila et al., 2022; Wang et al., 2022; Zhu Y. et al., 2022; Blanco-Rambo et al., 2023), and diets containing natural antioxidant compounds (Kumar et al., 2012; Maki, 2012; Smith et al., 2014; Barreto et al., 2017; Phipps et al., 2021; Tao et al., 2023; Tchekalarova and Tzoneva, 2023).

Most pathological conditions that provoke loss of cognitive abilities, including learning and memory deficits, can be triggered or exacerbated by aging (Murman, 2015). Brain atrophy during aging starts in the more plastic regions that are involved in learning and memory, such as the prefrontal cortex (PFC), the hippocampus (Hipp), the cingulate cortex (CC), and the entorhinal cortex (EC) to later extend throughout the brain (Blinkouskaya et al., 2021; Blinkouskaya and Weickenmeier, 2021).

### 6.1. Role of neuroinflammation in memory loss during aging

During aging, changes in the levels of neurotransmitters and hormones seem to make the brain more prone to suffer cerebrovascular accidents and white matter lesions, leading to memory impairment and dementia (Jett et al., 2022; Kanoni et al., 2022; Hogervorst et al., 2023; Kerstens et al., 2023). Cognitive impairment results from complex mechanisms affecting the brain’s function during aging. Mutations, genetic predisposition, biological clock, and a mix of extrinsic factors, including infectious diseases, chronic stress, and environmental toxins, enhance brain aging. These factors transform the glial cells to stop releasing neuroprotective factors, and release apoptotic cytokines, ultimately provoking energy deficits, oxidative stress, neuroinflammation, and neuronal loss.

Neuroinflammation is a common feature of most CNS diseases and a recognized mediator of brain dysfunction (Echeverria et al., 2016b). Neuroinflammation and oxidative stress accompany cognitive deterioration during aging by mechanisms altering the structure and function of glial cells (Echeverria, 2016). Glial cells, under pathological conditions, decrease the production of neurotrophic factors such as BDNF and show higher expression and release of cytokines. Cytokines are proteins that, when secreted, regulate the immune response and control cell growth, differentiation, and function. Cytokines released by macrophages, glial cells, and lymphocytes induce the innate immune response and a persistent cellular response by B cells and T cells. In addition, the binding of the cytokines to their receptors activates signaling pathways, enhancing the expression of genes that further increase the immune response (Jones and Thomsen, 2013). The cellular changes induced by inflammatory cytokines such as TNF-α and IL-1β, alter the connectivity of brain regions involved in information accurate acquisition and recall or interpretation of facts, leading to abnormal thinking and behaviors (Bowirrat, 2022). After TBI, infection, depression, and neurodegeneration, high levels of cytokines negatively affect brain homeostasis and plasticity, learning, memory, and emotional behavior, especially during aging (Ladak et al., 2019; Bourgognon and Cavanagh, 2020; Kalra et al., 2022).

Chronic neuroinflammation is common in most neurological or psychiatric conditions, leading to cognitive impairment and dementia (McGeer et al., 2000; McGeer and McGeer, 2010). Brain inflammation, oxidative stress, and energy deficits co-occur and can result from exposure to neurotoxins, physical and mental trauma, abnormal protein aggregates, stress hormones altering neurotransmission, and autoimmune reactions.

**Figure 1:**
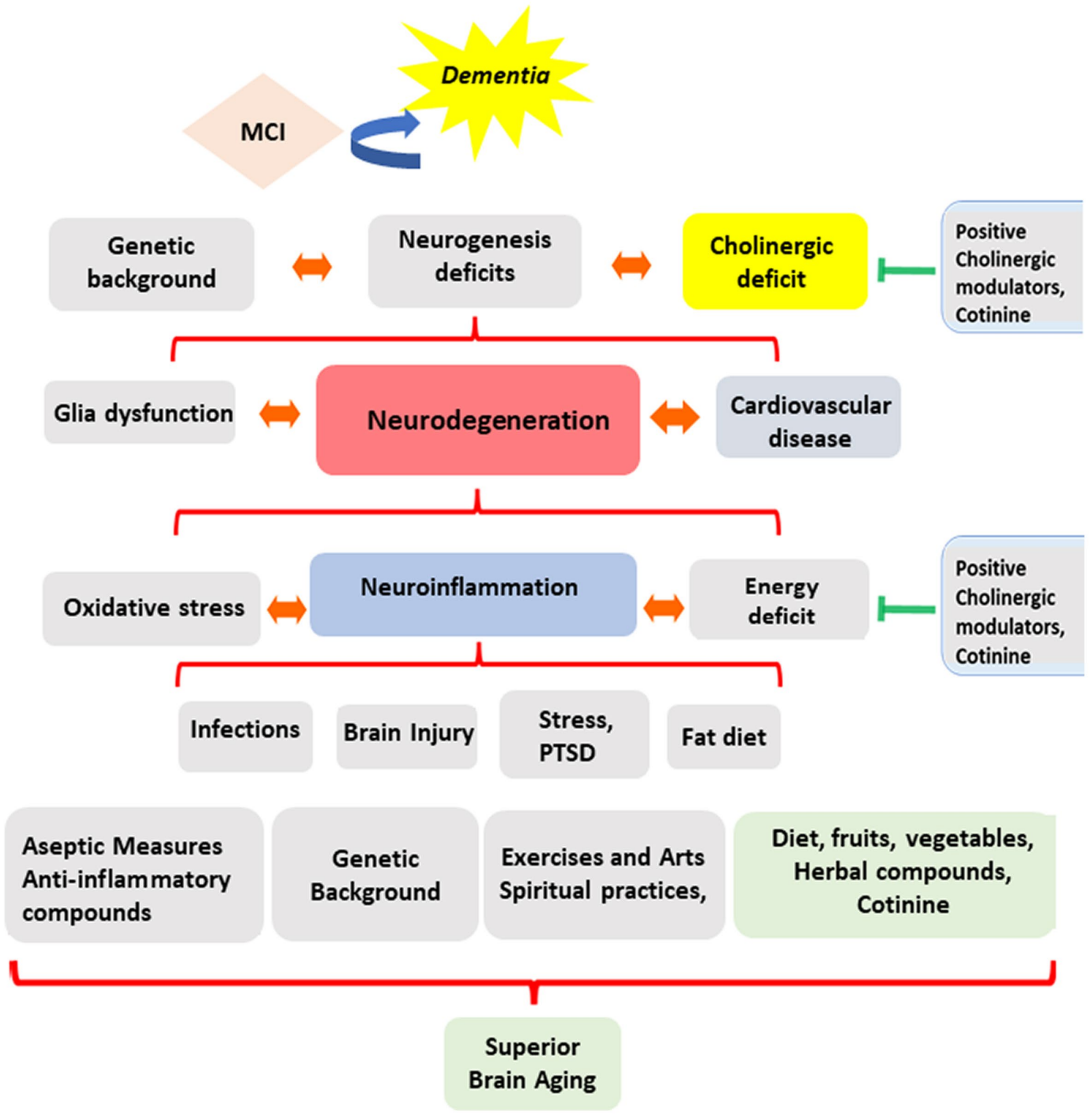
Evolution of cognitive abilities during aging. The diagram summarizes the complexity of the factors influencing the direction of mental changes during aging. These factors include experiencing inflammatory diseases, a person’s lifestyle, genetic background, diet, exercise habits, religious practices, and environmental, social, and personal stress factors affecting brain function.

A complex combination of genetic factors, life events, and lifestyles triggers these pathological processes. Mental triggers include traumatic events, depression (Gu et al., 2022), physical stressors such as radiotherapy (Gu et al., 2022; Gutierrez-Quintana et al., 2022), traumatic brain injury, viral or bacterial infections (Akinci et al., 2022; Francis et al., 2023), and exposure to toxins such as toxic heavy metals (Mani et al., 2022; Xu et al., 2022), insecticides, chemotherapy agents, medicaments, and a fatty diet. A recent study showed that neuroinflammation could also be associated with epigenetic changes induced by Posttraumatic Stress Disorder (PTSD) (Wolf et al., 2018) (see Figure 1).

## 7. Targeting adult neurogenesis to enhance memory abilities using positive modulators of the nAChRs

The CNS controls all body processes, from thinking, movement, and memory to regulating body temperature and hormone secretion. Therefore, one of the most exciting questions in medicine is why neurons have a limited regenerative capacity compared to other body parts. The CNS’s limited regenerative ability may help prevent inadequate integration of new neurons in a complex system, preventing disorganized behavior and altered perception that may endanger the organism’s survival. Almost 60 years have passed since the first reports suggesting that the mammalian brain could regenerate through the proliferation and differentiation of adult neural stem cells (Brett et al., 2021; Lippa et al., 2021; Steward et al., 2022).

Adult Neurogenesis is a lifelong process by which neural stem cells (NSCs) differentiate, mature, and integrate into local circuits (Aimone et al., 2014). Currently, two points of Neurogenesis in the brain are well known: the subventricular zone (SVZ), adjacent to the lateral ventricles, and the subgranular zone (SGZ) of the dentate gyrus (DG) of the hippocampus (Altman and Das, 1966; Altman, 1969a,b; Altmann et al., 2019). In addition, according to some data, Neurogenesis can also occur in the hippocampus, cerebral cortex, striatum, amygdala, and substantia nigra after brain damage (Gould et al., 2000; Li et al., 2023; Woitke et al., 2023; Yang J.L. et al., 2023). For example, some of the developing cells from the SVZ migrate as neuroblasts to the olfactory bulb, where they develop into olfactory interneurons (Doetsch et al., 1997, 1999; Doetsch and Scharff, 2001; Doetsch and Hen, 2005). In addition, part of these immature neuronal cells migrates to areas of the cerebral cortex where they may participate in cell repair or renewal (Parent, 2002). Similarly, newly formed cells migrate into the granular cell layer transforming into fully integrated mature dentate granular neurons (Kempermann et al., 2018). Significantly, newly generated DG granular neurons form synapses and attach axons to their correct part of the CA3 region, generating action potentials and functional synaptic inputs like mature DG neurons (Hastings and Gould, 1999).

According to some quantitative studies of the neurogenesis level in rodents, it is pretty high. For example, rat dentate gyrus produces up to 270,000 new cells per month, up to a quarter of the total number of granule cells (Cameron and McKay, 2001; Cameron and Glover, 2015). It is supposed that this level of new cell addition will undoubtedly change the function of the network. Indeed, numerous evidence supports the involvement of adult hippocampal Neurogenesis in learning and memory (Clelland et al., 2009). Fortunately, physical activity Increases the generation of new neurons, accelerates spatial learning, and enhances long-term potentiation (van Praag et al., 1999).

Conversely, reduced Neurogenesis in the hippocampus as a result of antimitotic drugs, radiation, or genetic manipulation impairs hippocampal function (Shors et al., 2001), long-term spatial memory (Rola et al., 2004; Snyder et al., 2005), and contextual fear conditioning and environmental enrichment (Meshi et al., 2006; Saxe et al., 2006). Compared to hippocampal Neurogenesis, the function of SVZ-olfactory Neurogenesis is less well-defined. However, a few studies have shown that new neurons in the olfactory bulb play a significant role in the maintenance of olfactory tissue and are involved in olfactory discrimination and olfactory perception functions (Kageyama et al., 2012).

### 7.1. Neurogenesis and aging

Neurogenesis happens, to a low extent, in specific brain regions and promoted by acetylcholine (ACh), a neurotransmitter pivotal in cognition (Madrid et al., 2021). Trying to understand how a small number of new hippocampal neurons can influence brain function, a study investigated adult neurogenesis across the lifespan. They found that chronic suppression of hippocampal Neurogenesis, as observed after exposure to chronic stress and aging, results in a hippocampal cholinergic deficit that parallels a progressive loss of working memory (Madrid et al., 2021). Also, they observed compensatory structural and functional changes in the cholinergic innervation of the hippocampal formation supported by new adult neurons that provide increased cholinergic inputs to the ventral hippocampus from ventrally projecting neurons that are recruited by the dorsal projection. They hypothesize that Neurogenesis supports memory by supporting the septohippocampal cholinergic circuit throughout the lifespan. They concluded that even aberrant new connections can be recruited to restore memory functions in aged subjects. Furthermore, neurogenesis in the hippocampus is critical to maintain social memory and can be by social factors Oxytocin, a hormone stimulated by social interaction, stimulates neurogenesis under common and stressful conditions and social isolation has the opposite effect (Leuner et al., 2012; Holmes, 2016). Neurogenesis occurs at a calculated rate of 1.75% per year (Spalding et al., 2013) and persists throughout aging (Boldrini et al., 2018; Tartt et al., 2018).

Some scientists in the field argued that there is insufficient evidence to sustain the existence of adult neurogenesis in humans, and that generation of neurons and their migration are limited to early childhood (Curtis et al., 2012; Kumar et al., 2019a, 2020). Despite these suggestions, the research on adult neurogenesis continues growing, holding great promise for brain rehabilitation, and helping to modifying stressful memories (Scott et al., 2021). Stimulating endogenous brain repair mechanisms would be more effective from an immunological and physiological point of view than using exogenous cell implantation approaches (Sun, 2014; Poulose et al., 2017; Scott et al., 2021). Opening opportunities stimulate scientists to study Neurogenesis in various aspects, including the search for controlling and modulating signals (Shabani et al., 2021). Other therapeutic approaches include the use of plant extracts, plant compounds or nutritional factors (functional food), neurotransmitters (serotonin, acetylcholine), sex hormones (estrogen and testosterone), glucocorticoids, and neuronal growth factors (NGF, BDNF) to target biochemical factors modulating neuronal cell proliferation or differentiation (Poulose et al., 2017; Setel et al., 2022; Shengkai et al., 2022; Zheng et al., 2022).

Current evidence suggests that the most promising factors to stimulate neurogenesis are the ones that positively modulate the cholinergic system, which has a vital role in adult neurogenesis (Harrist et al., 2004; Mohapel et al., 2005; John et al., 2015). We and others propose that acetylcholine receptors, especially the α7 and α4β2 subtypes, are the most promising targets to promote neurogenesis in the hippocampus (Harrist et al., 2004; Campbell et al., 2010; Hernandez-Miranda et al., 2010; John et al., 2015; Otto and Yakel, 2019; Webster et al., 2019). The α7nAChRs are expressed in embryonic and adult NSCs and play an essential role in activating its proliferation and differentiation, thus regulating hippocampal neurogenesis in a sex-dependent manner (Otto and Yakel, 2019). Furthermore, the nAChRs may influence Neurogenesis by inhibiting neuroinflammation and regulating fibroblast growth factor-1 (FGF-1) and FGF-2 signaling (Narla S. et al., 2013; Narla S.T. et al., 2013). The subgranular zone and the inner granular cell layer, which are neural stem cell niches, have rich cholinergic innervation and solid responses to cholinergic stimulation suggesting a role of the nAChRs in neurogenesis (Doetsch and Hen, 2005; Riquelme et al., 2008).

In addition, evidence showed that degeneration of the cholinergic system suppresses Neurogenesis in the hippocampus (Kotani et al., 2006; Ijomone and Nwoha, 2015). The survival and development of neurons in the hippocampus require Ca^2+^-dependent activation of the ERK and CREB by the nAChRs (Shabani et al., 2020; Shen et al., 2022). The enhancement of Neurogenesis involves the trafficking of α7nAChR, which requires increased phosphorylation of Protein Kinase C (PKC) and a decrease in the level of farnesyl pyrophosphate (Chen et al., 2018). In the brain, α7nAChR activation stimulates neuroprotection and neuronal survival by increasing Bcl-2 expression via the PI3K/Akt pathway (Kihara et al., 2001). Overall, current evidence suggests that partial agonists, antagonists, and positive modulators of the α7nAChRs can stimulate and inhibit adult neurogenesis. Thus, the positive impact of cotinine on neurogenesis can be explained at least partly by the positive modulation of the α7nAChRs.

## 8. Cognitive-enhancing effects of cotinine in mice models of PTSD, aging, and Alzheimer’s disease

We and others have shown evidence suggesting that hippocampal cholinergic receptors are fundamental to regulating the extinction of fear memories showing that antagonists of the nAChRs or mAChRs impair fear memory extension when directly administered in the hippocampus using stereotaxic techniques (Oliveros-Matus et al., 2020; Rosa et al., 2023).

Cotinine is a member of a family of >60 alkaloid derivatives of nicotine and its primary metabolite (Grizzell and Echeverria, 2015). Cotinine is produced by the oxidation of nicotine by the Cytochrome P450 and aldehyde oxidases and further modified by other metabolic enzymes to be eliminated in the urine (Siu and Tyndale, 2007; Zhou et al., 2010). As a result, cotinine accumulates in the brain and has a better safety profile than nicotine and its derivatives, having minimal side effects in humans (Grizzell and Echeverria, 2015).

In animals, Cotinine has been administered via oral, intranasal, intravenously, and by intramuscular or intracerebral injection showing strong memory and mood-enhancing effects under normal and pathological conditions in rodents (Dar et al., 1993; Schroff et al., 2000; Zeitlin et al., 2012; Grizzell et al., 2014a; Patel et al., 2014; Wildeboer-Andrud et al., 2014; Grizzell et al., 2017; Perez-Urrutia et al., 2017; Alvarez-Ricartes et al., 2018a; Mendoza et al., 2018b; Oliveros-Matus et al., 2020), monkeys (Terry et al., 2005; O'Leary et al., 2008), humans, and zebrafish (Boiangiu et al., 2021). In addition, Cotinine has shown unprecedented transversal benefits for cognitive abilities in animal models of mental illnesses or developmental, including schizophrenia (Moran, 2012; Echeverria et al., 2016a), autism (Wang et al., 2015), depression (Grizzell et al., 2014b; Patel et al., 2014; Mendoza et al., 2018b), chronic stress (Alvarez-Ricartes et al., 2018b), anxiety (Barreto et al., 2017), PTSD (Barreto et al., 2015), AD (Patel et al., 2014; Echeverria et al., 2016a, 2017), aging (Terry et al., 2005; Moran, 2012) and cellular models of PD (Soto-Otero et al., 2002; O'Leary et al., 2008; Barreto et al., 2014). Furthermore, Cotinine improved cognitive abilities and mood with no significant adverse events in rodents, monkeys, fish, and humans (Hatsukami et al., 1992, 1997, 1998a,b; Keenan et al., 1994).

Despite various studies showing the safety of cotinine in humans (Hatsukami et al., 1997; Terry et al., 2005), no clinical studies of Cotinine as a treatment for psychiatric and neurological conditions have been performed. Nevertheless, last year a nasal spray containing a nicotinic Cholinergic Agonists Mixture (CAM), that included Cotinine, anatabine, and nicotine, was tested as a therapy in 80 adult Colombian patients with confirmed SARS-CoV-2 (Sharma and Mehan, 2021). The results showed that in these critical patients, after 2 weeks of treatment, the patients showed a statistically and clinically significant reduction of five symptoms, including dyspnea, cough, general malaise, muscle fatigue, and cephalea (Sharma and Mehan, 2021). In this and other conditions alleviated by Cotinine, there was an underlying deficit in nAChRs, neuroinflammation, and oxidative stress (Tan et al., 2021).

### 8.1. Current therapies for cognitive impairment in AD

Currently, the amyloid hypothesis stating that Aβ aggregation is the leading cause of AD (McGeer and McGeer, 2013) is subjected to severe scrutiny, mainly because the therapies directed to reduce the senile Aβ plaques or the peptide aggregation have failed or proven minimally effective (Hilund-Carlsen et al., 2022). In fact, for long time, it has been recognized that brains with significant amyloid Aβ burden can still function well when neuroinflammation is under control (Echeverria et al., 2009). The abnormalities of the microtubule protein Tau have proven to be better at predicting brain atrophy and cognitive deficits in AD (Malpetti et al., 2022). Although, considerable evidence supports the view that aggregated forms of Aβ are part of the pathological equation (Hill, 2022). The Aβ peptide induces oxidative stress, tau hyperphosphorylation, synaptic plasticity deficits, and neuroinflammation (Choi and Gandhi, 2018; Rejc et al., 2022). To overcome similar paradoxes, new diagnostic tools such as the 18F-THK5351, a PET tau tracer that binds to the astroglial monoamine oxidase B, are being characterized to detect tau aggregation and neuroinflammation simultaneously (Imabayashi et al., 2022).

The drugs approved by the US Food and Drug Administration (FDA) to treat cognitive impairment include acetylcholinesterase inhibitors (AChEI), donepezil, galantamine, and rivastigmine, the NMDA receptor antagonist memantine and a new immunotherapeutic treatment; these drugs delay the progression of the disease but do not stop its progression (Porsteinsson et al., 2008; Kavanagh et al., 2011; Tayeb et al., 2012). The treatment for immunotherapy against Aβ peptides aggregation with aducanumab has been controversial, being one of the arguments that quantification of amyloid deposition by PET was imprecise due to the lack of specificity of the PET imaging probe (Hilund-Carlsen et al., 2022).

Another approach would be using positive allosteric modulators (PAM) to enhance the nAChRs activation in brain cells, to increase brain plasticity and Neurogenesis, and prevent the activation of glial cells and peripheric immune cells, such as macrophages and T cells, that reach the brain. Altogether this strategy would prevent neuroinflammation, neurodegeneration, and memory loss during aging and pathological conditions affecting the brain (Echeverria et al., 2016a).

Clinical trials have tested many pro-cholinergic compounds showing adverse side effects or lower efficacy. However, there are reasons to believe that these failures were because these therapies primarily involved agonists of these receptors, many of which rapidly desensitize the nAChRs eliciting undesired side effects. In addition, these compounds did not prevent tau hyperphosphorylation and Amyloid β peptide (Aβ) aggregation in the brains of people with dementia (Echeverria et al., 2016b).

Galantamine Hs shown to be superior to donepezil, improving cognitive impairment, most likely because, in addition to inhibiting ACh degradation, it is a PAM of the α7nAChR and inhibits the toll-like receptors (TLRs) (Bagaitkar et al., 2012). Other clinical studies investigating galantamine plus memantine did not show a superior effect of this combination in improving memory than galantamine as a single therapy in AD (Thancharoen et al., 2019). Galantamine and memantine increased the circulating level of the brain-derived neurotrophic factor (BDNF), most likely through the modulation/activation of the α7nAChRs and NMDARs, which can explain at least in part its pro-cognitive and neuroprotective effects (Bozon et al., 2003; Josselyn et al., 2004; Josselyn and Nguyen, 2005).

Based on our results investigating the effect of cotinine on learning and memory consolidation and extinction *in vivo* and the response of the human a7nAChR to ACh or nicotine, our team was the first to propose that a deficit in the expression and activity of the α7nAChRs was vital in promoting cognitive deficits and brain neurodegeneration during aging and after traumatic experiences (Sabandal et al., 2022). After more than a decade, all new evidence supports the idea that Cotinine acting on the nAChRs present in neurons, immune and glial cells prevents the release of inflammatory factors and neuroinflammation. Also, by modulating these receptors in neurons, endothelial cells, and neuronal precursor cells, cotinine can enhance Neurogenesis and strengthens the synapsis in the brain (Valles et al., 2014). Recent studies have shown that an enhanced risk of PTSD and AD during aging relates to differences in susceptibility genes involved in immune function, neuroinflammation, and stress responses. This evidence further supports the view that cotinine controlling neuroinflammation can prevent aging-induced cognitive decline (Abbatecola et al., 2011; Sabbagh et al., 2014; Criado-Marrero et al., 2018; Sawyer et al., 2022; Bathini et al., 2023).

## 9. Conclusion

Based on the evidence described in this review, it is reasonable to propose that correcting the nicotinic receptor deficits during aging may normalize neurogenesis, prevent the abnormal aggregation of Tau, APP, and α-synuclein and consequently, neuroinflammation, and neurodegeneration. The prevention of the accumulation of these pathogenic protein aggregates may delay or completely revert cognitive decline during aging (Maelicke, 2000; Coyle and Kershaw, 2001; Jurgensen and Ferreira, 2010; Toyohara and Hashimoto, 2010; Geerts, 2012; Echeverria et al., 2016b).

## Author contributions

All authors listed have made a substantial, direct, and intellectual contribution to the work and approved it for publication.

## Funding

This study was supported by the grant Fondecyt, ANID 1190264 (VE and AI).

## Conflict of interest

VE is the inventor of five patents granted for the use of cotinine for posttraumatic stress disorder, Alzheimer’s disease, and other neurological and psychiatric conditions.

The remaining authors declare that the research was conducted in the absence of any commercial or financial relationships that could be construed as a potential conflict of interest.

## Publisher’s note

All claims expressed in this article are solely those of the authors and do not necessarily represent those of their affiliated organizations, or those of the publisher, the editors and the reviewers. Any product that may be evaluated in this article, or claim that may be made by its manufacturer, is not guaranteed or endorsed by the publisher.
